# A Novel Immunobiotics *Bacteroides dorei* Ameliorates Influenza Virus Infection in Mice

**DOI:** 10.3389/fimmu.2021.828887

**Published:** 2022-01-26

**Authors:** Liqiong Song, Yuanming Huang, Guoxing Liu, Xianping Li, Yuchun Xiao, Chang Liu, Yue Zhang, Jintong Li, Jianguo Xu, Shan Lu, Zhihong Ren

**Affiliations:** ^1^ State Key Laboratory for Infectious Disease Prevention and Control, National Institute for Communicable Disease Control and Prevention, Chinese Center for Disease Control and Prevention, Research Units of Discovery of Unknown Bacteria and Function (2018 RU010), Chinese Academy of Medical Sciences, Beijing, China; ^2^ Respiratory Department, Beijing University of Chinese Medicine Third Affiliated Hospital, Beijing, China; ^3^ Traditional Chinese Medicine Department, Linwei Liu Zunji Clinic of Traditional Chinese Medicine, Weinan, China; ^4^ Respiratory Department, Dongzhimen Hospital, Beijing University of Chinese Medicine, Beijing, China

**Keywords:** *B. dorei*, influenza, interferon, gut microbiota, cytokines

## Abstract

**Objective:**

Probiotics can modulate immune responses to resist influenza infection. This study aims to evaluate the anti-viral efficacy of *B. dorei*.

**Methods:**

C57BL/6J mice were infected with influenza virus together with treatment of PBS vehicle, *B. dorei*, or oseltamivir respectively. Anti-influenza potency of *B. dorei* and the underlying mechanism were determined by measuring survival rate, lung viral load and pathology, gene expression and production of cytokines and chemokines, and analysis of gut microbiota.

**Results:**

Administration of *B. dorei* increased (by 30%) the survival of influenza-infected mice, and improved their weight loss, lung pathology, lung index, and colon length compared to the vehicle control group. *B. dorei* treatment reduced (by 61%) the viral load of lung tissue and increased expression of type 1 interferon more rapidly at day 3 postinfection. At day 7 postinfection, *B.* dorei-treated mice showed lower local (lung) and systemic (serum) levels of interferon and several proinflammatory cytokines or chemokines (IL-1β, IL-6, TNF-α, IL-10, MCP-1 and IP-10) with a efficacy comparable to oseltamivi treatment. *B. dorei* treatment also altered gut microbiota as indicated by increased levels of Bacteroides, Prevotella, and Lactobacillus and decreased levels of Escherichia, Shigella, and Parabacteroides.

**Conclusion:**

*B. dorei* has anti-influenza effect. Its working mechanisms involve promoting earlier interferon expression and down-regulating both local and systemic inflammatory response. *B. dorei* changes the composition of gut microbiota, which may also contribute to its beneficial effects.

## Introduction

Influenza virus can cause seasonal epidemics and sometimes the global pandemics, and thus it has become a serious public health burden (1). Effective vaccination is the best way to prevent communicable diseases. Although influenza vaccine has been used widely for many years, it cannot significantly prevent the influenza epidemic as in the case of measles vaccine for measles epidemic, which is mainly due to the high mutation of the influenza virus (2). The World Health Organization has established a global influenza surveillance network and recommends the appropriate dominant influenza virus strains annually to vaccine manufacturers based on the monitoring results. However, there are still frequent mismatches occurred between influenza vaccines and the epidemic virus strains, which diminishes the protection of the vaccination. There are some anti-viral drugs available such as Oseltamivir and Zanamivir (3, 4), however, the drug resistance after long-term use limits their value for influenza treatment. Therefore, developing alternative strategies of preventing influenza is warranted.

Probiotics are defined as”live microorganisms that, when administered in adequate amount, confer a health benefit to the host”(FAO/WHO: Report of a joint FAO/WHO expert consultation). Probiotics can benefit human health by maintaining intestinal microecology and modulating immune function. Some probiotics have shown to have antiviral potency against influenza virus, and such probiotics include *Lactobacillus gasseri* (5, 6), *Lactobacillus plantarum* (1, 7), *Lactobacillus rhamnosus* (8, 9), *Lactobacillus acidophilus* (10), *Lactobacillus paracasei* (11), *Bifidobacterium longum* (12–14), and *Bacillus subtilis* (15). Moreover, probiotics can be used as functional food for long-term without obvious adverse effects as seen in the conventional anti-influenza drugs. Therefore, the probiotics with anti-influenza activity may serve as an important adjuvant therapy to prevent the influenza epidemic together with influenza vaccines and anti-influenza drugs (16, 17).

The potential anti-influenza probiotics reported thus far are mainly *Lactobacillus* and *Bifidobacterium* genus with varied potency. It has been shown that probiotics can enhance immune response to infection or alleviate trauma through their metabolites (18, 19). Flavonoids have beneficial effects on human health based on their anti-inflammatory, antioxidant, vasodilatory, anti-carcinogenic, and antibacterial properties (20). Human gut microbiota, such as *Clostridium scindens*, *Clostridium orbiscindens* (21), *Eubacterium desmolans*, and *Eubacterium ramulus* (22), can convert flavonoids. The anaerobic *Clostridium orbiscindens* is reported to prevent influenza by degrading flavonoids to generate desaminotyrosine (DAT), a microbial metabolite which can augment type 1 interferon signaling. DAT-induced type 1 interferon in the gut then acts on intrinsic immune cells in the lungs to suppress virus replication *via* blood circulation (23). Therefore, it is possible that the gut commensal bacteria that metabolize flavonoids may prevent influenza through promoting type 1 interferon.

In this study, we screened 48 strains of bacteria based on their capacity of metabolizing quercetin and selected the novel probiotic candidate strain *Bacteroides dorei* XR 2020 *(B. dorei)* to assess its anti-influenza activity. *B. dorei* strain JCM 13471 was first isolated from human feces in 2006 (24). The functions of *B. dorei* reported include inhibition of the growth of *C. difficile* (25, 26) and suppression of LPS production and atherosclerosis (27). Recently, it was reported that the counts of *B. dorei* were negatively correlated with SARS-CoV-2 viral load in fecal samples of hospitalized COVID-19 patients (28, 29). We hypothesized that *B. dorei* may exert anti-influenza activity and conducted the current study to test this hypothesis.

## Materials And Methods

### Bacterial and Virus Culture

A total of 48 bacteria were isolated from fermented food or stool specimens of healthy human and identified utilizing 16S rRNA sequencing in our laboratory. The probiotic candidate *B. dorei* XR2020 was isolated from stool specimen of a healthy human using brain heart immersion (BHI) agar medium supplemented with 5% defibrotic sheep blood in a 5% CO2 incubator at 37°C for 48 hours (anaerobic incubator: Electrotek Scientific UK). An anaerobic bacteria, Clostridium orbiscindens (ATCC 49531), was purchased from Zhongyuan Inc. and used as the positive control strain for degrading quercetin.

Influenza A virus A/Puerto Rico/8/34 mouse-lung-adaptive strain (PR8) was kindly provided by the Chinese National Influenza Center, National Institute for Viral Disease Control and Prevention, Chinese Center for Disease Control and Prevention.

PR8 was reproduced with Madin-Darby canine kidney (MDCK) cells and quantified using a plaque assay as previously described (29). Briefly, MDCK cells were cultured in 6-well plates with Dulbecco’s Modified Eagle Medium (DMEM) supplemented with 10% Fatal Bovine Serum (FBS). Then 0.2 ml of the 1000-fold dilutions PR8 was inoculated in monolayers of MDCK cells in 6-well plates, incubated for 1.5 h, and overlaid with 4 ml of 1% methylcellulose. After incubation in an incubator with 5% CO2 atmosphere at 37°C for 3 days, the plaques in each well were counted using crystal violet-staining, and the viral titer was calculated as plaque forming unit (PFU) per ml.

### Animal Infection With Influenza Virus

All procedures of this animal study were approved and performed in accordance with the Welfare and Ethical Inspection in Animal Experimentation Committee at the Chinese CDC.

Female C57BL/6J mice (6 weeks, 13-15g) purchased from Beijing Vital River Laboratory Animal Technology Co., Ltd (Certificate No.: SCXK(Beijing) 2012-0001), China) were housed in the Experiment Animal Center of China Center for Disease Control and Prevention (Certificate No.: SYXK(Beijing) 2017-0021).

**Figure 1:**
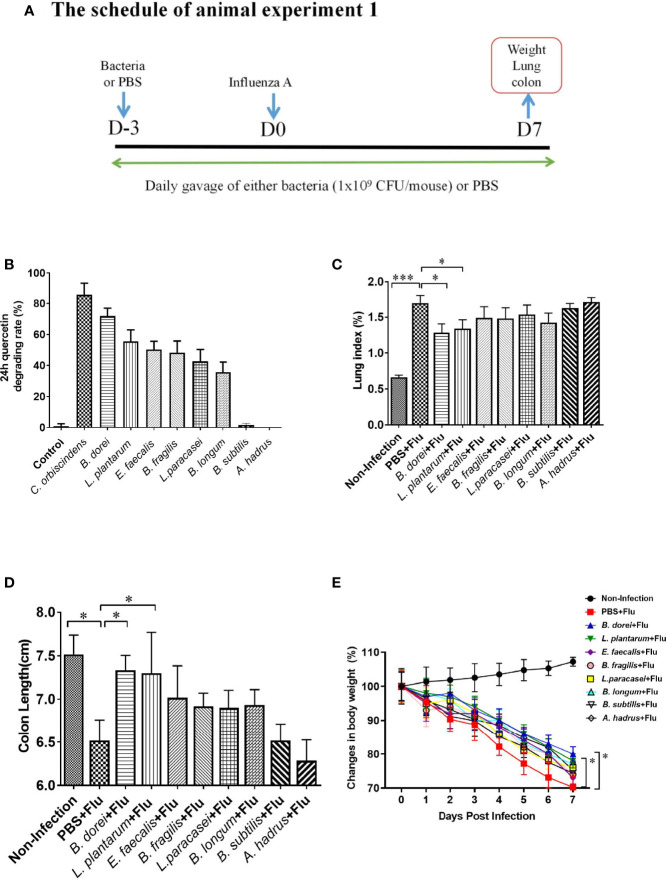
Comparison of eight probiotics candidates for quercetin-degrading and anti-influenza efficacy. **(A)** Study design of animal experiment 1. Sixty 6-week old female C57BL/6J mice were randomly allocated to 10 groups (6 mice/group): control group (negative control), *Clostridium orbiscindens* group (positive control), *Bacteroides dorei* group (*B. dorei*), *Lactobacillus plantarum* group (*L. plantarum)*, *Enterococcus faecalis* group (*E. faecalis*), *Bacteroides fragilis* group (*B. fragilis*), *Lactobacillus paracasei* group (*L. paracasei*), *Bifidobacterium longum* group (*B. longum*), *Bacillus subtilis* group (*B.Subtilis*), *Anaerostipes hadrus* group (*A. hadrus*); **(B)** Comparison of eight probiotics for quercetin-degrading efficacy; **(C)** Comparison of the lung index; **(D)** Comparison of the colon length; **(E)** Dynamic trend in body weight after influenza infection. The data in panel **(B)** are means ± SD of three independent experiments. The data in panels **(C–E)** are from one of three independent experiments. *p < 0.05, ***p < 0.001.

Mice were housed with 5 per cage and had free access to water and food under a 12-hour light cycle. Three animal experiments were performed in this study. The aim of experiment one was to screen and select the best anti-influenza strain from 8 candidate strains. The mice were randomly allocated to 10 groups (6 mice per group) after 7-day adaptation period. Except the non-infection mice, all mice were anesthetized with intraperitoneal injection of 1% Pentobarbital and then inoculated intranasally with 540 PFU PR8 (nonlethal dose) on the infection day (day 0). The infected mice were treated with 200 µL PBS (control) or different candidate strains (1×10^9^ CFU per mouse) once daily by gavage starting 3 days prior to infection for 10 consecutive days throughout the experiment (**Figure 1A**). All mice were monitored daily for body weight and euthanized on day 7 post infection (p.i.). The colon and whole lung were collected to determine the colon length and lung index (Lung index is the percent ratio of lung weight to body weight of mice, Lung index = lung weight of mice (g)/body weight of mice (g) × 100%). The lung index is commonly used in respiratory infection models; it usually elevates in the infected lungs due to infection-induced inflammatory response and increased lung weight, as well as the substantial body weight loss (30).

The aim of experiment two was to determine the optimal time point for administering the selected candidate strain (*B. dorei*) determined in the experiment one described above. Twenty-four mice (6 mice per group) were randomly divided to four groups: Non-infection group, PBS+Flu group, Pre-treated-*B. dorei*+Flu group, Post-treated-*B. dorei*+Flu group. Except those in non-infection group, all mice were anesthetized and injected intraperitoneally with 1% Pentobarbital and then inoculated intranasally with 540 PFU PR8 (nonlethal dose) on the infection day (day 0).

The Pre-treated-*B. dorei*+Flu group and PBS+Flu group were administered with 200 µL *B. dorei* (1×10^9^ CFU) or PBS by gavage once daily starting 3 days prior to infection for 10 consecutive days. The Post-treated *B. dorei*+Flu group was administered with equal amount of *B. dorei* on 2 h p.i. till the end of experiment. All mice were euthanized and the whole lungs and colon were obtained for measuring lung index and colon length on day 7 p.i. (**Supplementary Figures S1A–E**).

**Figure 2:**
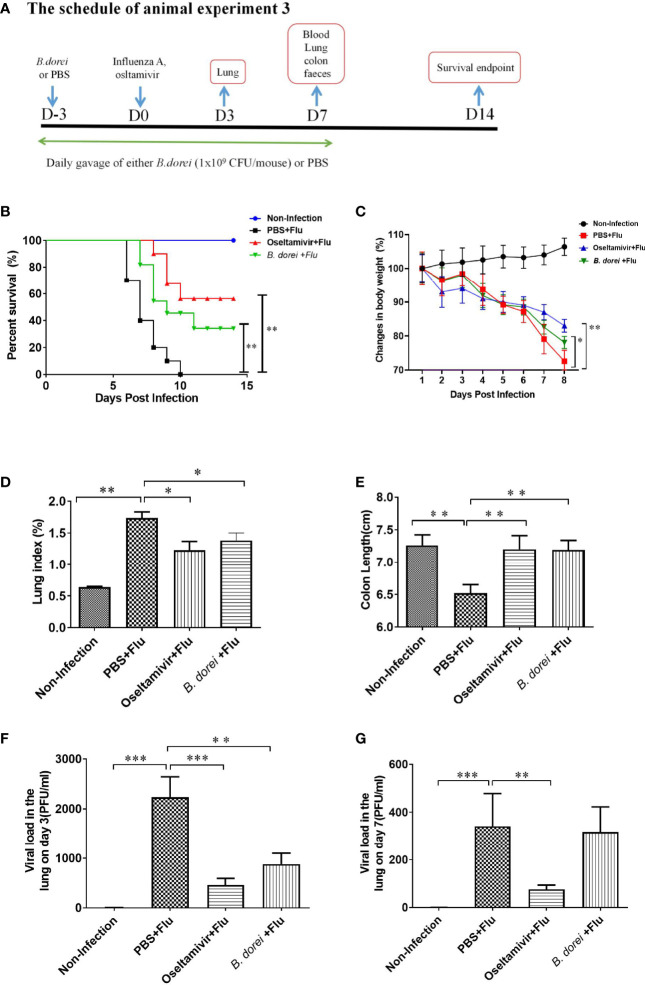
Protective effect of *B. dorei* pre- treatment against influenza infection. **(A)** shows the schedule of animal experiment 3. One experiment was conducted for survival analysis. Forty 6-week-old female C57BL/6J mice were randomly allocated to 4 groups with 10 mice per group: Non-Infection group (Control group), PBS+Flu group (PBS+influenza virus group), *B. dorei*+Flu group (*B. dorei*+influenza virus group), oseltamivir+Flu group (oseltamivir+influenza virus group). Another experiment focused on weight loss, anti-inflammation and virus load. Eighty mice were randomly allocated to the above mentioned 4 groups with 20 mice per group. **(B)** shows the survivals 14 days post infection of four groups in animal experiment; **(C)** shows the dynamic trend in the body weight of the mice in each group after infection; **(D)** shows the lung index of the mice in each group at day 7 p.i.; **(E)** shows the colon length of the mice in each group at day 7p.i; **(F)** shows the viral load in lung tissue day 3 post infection; **(G)** shows the viral load in lung tissue day 7 post infection. The data in panels **(B–C)** are obtained from one of three independent experiments. The data in panels **(D–G)** are shown as mean ± standard deviation from three independent experiments. *p < 0.05, **p < 0.01, ***p < 0.001.

The animal experiment three was conducted to further evaluate the protection efficacy of *B. dorei* against influenza infection. A total of 120 mice were randomly divided into four groups (10 mice per group): Non-infection group, PBS+Flu group, Oseltamivir+Flu group, and *B.dorei*+Flu group. The *B. dorei*+Flu group was administered with 200 µL *B. dorei* (1×10^9^ CFU) by gavage once daily starting 3 days prior to infection for 10 consecutive days. The Oseltamivir+Flu group was administered with equal amount of Oseltamivir (19.19mg/kg) by gavage once daily on 2 h p.i. till the end of experiment. All mice except non-infection mice were anesthetized and injected intraperitoneally with 1% Pentobarbital and then inoculated intranasally with 540 PFU PR8 (nonlethal dose) on infection day (day 0). A subset of mice (n=10) were sacrificed on day 3 p.i. and whole lungs were obtained for measuring viral load and expression of cytokines and chemokines. Another subset of mice (n=10) were monitored for body weight daily and euthanized on day 7 p.i. The whole lungs were collected for measuring lung index, viral load, cytokines and chemokines, and lung pathology. The colon and feces were collected to determine the colon length and gut microbiota by 16 sRNA sequencing. To determine the effect of *B. dorei* on survival rate of mice infected by influenza, ten mice of each group were challenged with a lethal dose (850 PFU) of PR8 (day 0), and observed daily for up to 14 days p.i. (**Figure 2A**).

### Assay for Quercetin Degradation

Five mg of quercetin was dissolved in 3mL DMSO, and this quercetin solution was then added to 47 mL BHI medium to make working solution of quercetin. 1×10^7^ CFU of different probiotic strains and *Clostridium orbiscindens* (ATCC 49531) (positive control) in 0.5 ml PBS were added into 4.5 ml (Brain Heart) BHI medium containing 0.1mg/ml quercetin and cultured in an anaerobic incubator at 37°C for 24 h. After centrifugation at 8000 rpm for 5 min, 100 µl supernatant was collected and mixed with equal volume of 1% AlCl_3_ (dissolved in methanol) and incubated at room temperature for 5 min. The optical density (OD) value was measured with a microplate reader at 405 nm. BHI was used as a negative control. The BHI medium with 0.1 mg/ml quercetin was 2-fold serially diluted to make graded standard solutions.

### Measurement of Influenza Viral Load in Lung Tissues

The lung tissues from infected mice were homogenized using a tissue grinder (Qiagen, Germany, Model number: Tissuelyser II) and centrifuged at 12000 rpm for 10 min to obtain supernatant. Influenza virus RNA in the supernatant was extracted using QIAamp Viral RNA Mini Kit (QIAGEN GmbH, Lot.: 163014099). The load of H1N1 influenza virus in lung was determined using influenza A virus nucleic acid test kit (Jiangsu Hechuang Biotechnology Co., Ltd.) according to the manufacturer’s instructions on an ABI 7500 Real Time PCR System (ABI, United States).

### Lung Histopathological Examination

Lung tissues were collected and fixed in 10% formalin solution for 24 hours, then tissue was embedded in paraffin and sliced into 5 μm thick sections, which was stained with hematoxylin and eosin (H/E). The sections were observed under light microscopes by two experienced pathologists. Histopathological changes were scored as 1 to 5 according to the lung histopathological scoring criteria described by Roderick et al. (31).

### Analysis of Cytokine and Chemokine Gene Expression in Lung

Total RNA of lung tissue was extracted using Trizol according to the manufacturer’s instructions. The obtained RNA samples were quantified by measuring the absorbance at 260 nm wavelength and diluted to 500 ng/μL for Real-time PCR. RNA was first reverse transcribed into cDNA as the template for the following amplification. A total of 10 proinflammatory cytokines and chemokines, including IL-1β, TNF-α, IL-6, IL-10, IL-12, IFN-α, IFN-β, IFN-γ, MCP-1, and IP-10, were measured by qRT-PCR (TB GreenTM Premix reagent). A total 20-μL reaction, containing 2 μL cDNA, 10 μL TB Green Premix Ex Taq™ II, 0.8 μL upstream and downstream primers (10 μmol/L), 0.4 μL ROX Reference DyeII(50×), was performed on an ABI 7500 Real-Time PCR System under the following conditions: pre-denature at 95°C 30s, followed by 40 cycles of amplification (denatured at 95°C for 3s, annealed and extended at 60°C for 30s) and melting curves analysis from 60°C to 95°C. Relative quantity of the target gene was calculated using the 2^-ΔΔCt^ method and β-actin was used as an internal control. The sequences of qRT-PCR primers were listed in **Table 1**.

**Table 1:** List of primers used in the qRT-PCR assay for cytokines, chemokines, and IFNs.

Primer	Sequence (5’ to 3’)
TNF-α-F	CTGTAGCCCACGTCGTAGC
TNF-α-R	TTGAGATCCATGCCGTTG
IL-1β-F	GGACATGAGCACCTTCTTTTCC
IL-1β-R	GTGCCGTCTTTCATTACACAGG
IL-6-F	ACAAGTCGGAGGCTTAATTACACAT
IL-6-R	TTGCCATTGCACAACTCTTTTC
IL-10-F	GGTTGCCAAGCCTTATCGGA
IL-10-R	ACCTGCTCCACTGCCTTGCT
IL-12-F	GGTCACACTGGACCAAAGGGACTATG
IL-12-R	ATTCTGCTGCCGTGCTTCCAAC
IFN-α-F	GTGAGGAAATACTTCCACAGGATCAC
IFN-α-R	TCTCCAGACTTCTGCTCTGACCA
IFN-β-F	CAGCTCCAAGAAAGGACGAAC
IFN-β-R	GGCAGTGTAACTCTTCTGCAT
IFN-γ-F	GCCACGGCACAGTCATTGA
IFN-γ-R	TGCTGATGGCCTGATTGTCTT
CCl2/MCP-F	TTAAAAACCTGGATCGGAACCAA
CCl2/MCP-R	GCATTAGCTTCAGATTTACGGGT
CXCL10/IP-10-F	CCAAGTGCTGCCGTCATTTTC
CXCL10/IP-10-R	GGCTCGCAGGGATGATTTCAA

### Measurement of Cytokines in Serum and Lung Tissue

A total of 8 cytokines and chemokines were measured using a LabEx Biomarker Mouse 8-Plex kit (LXSAMSM-8, RnD) on Luminex 200 following the manufacturers’ instructions, including TNF-α, IL-1β, IL-6, IL-10, IL-12, IFN-γ, MCP-1 and IP-10 from serum and lung tissue homogenates.

### High-Throughput Sequencing of 16 S rRNA Genes of Gut Microbiota

Total genome DNA in feces sample was extracted using the QIAamp DNA Stool Mini Kit (Qiagen, Germany) according to the manufacturer’s instructions. DNA concentration was determined using Qubit dsDNA HS Assay Kit (Invitrogen), and the integrity of DNA was assessed on 1% agarose. V3 and V4 hypervariable regions of 16S rRNA were selected and amplified using primer pairs: Forward primer (5’-CCTACGGGNGGCWGCAG-3’) and Reverse primer (5’-GACTACHVGGGTATCTAATCC-3’). Sequencing of the PCR amplicons and quality control of the raw data were performed on the Illumina MiSeq PE250 platform. Overlapping paired-end reads from the original DNA fragments were merged using FLASH (v1.2.11). High-quality reads were grouped into operational taxonomic units (OTUs) by USEARCH (v7.0.1090) with a 97% threshold. The Shannon_e index and principal co-ordinates analysis (PCoA) were calculated and visualized by R software (v3.1.1). Key differential OTUs were identified by Linear discriminant analysis (LDA) effect size (LEfSe) analysis (http://huttenhower.sph.harvard.edu/galaxy/) based on nonparametric factorial Kruskal-Wallis and Wilcoxon rank sum tests with significance level set at < 0.05. The threshold on logarithmic LDA score for discriminative features was set at 2.0. The original data can be downloaded from the NCBI SRA database (PRJNA769975).

### Statistical Analysis

Continuous variables were expressed as mean ± standard deviation (SD), category variables were expressed as n (%). Between two group comparisons were conducted using Student *t* test or Mann-Whitney rank sum test when whichever was appropriate. Comparisons among ≥3 groups were conducted using one-way analysis of variance (ANOVA) or Kruskal-Wallis rank-sum when whichever was appropriate. Multiple comparisons were conducted using least significance difference t (LSD-t) test or Kruskal-Wallis rank-sum test with Bonferroni correction when whichever was appropriate. Survival of mice in different groups were estimated using Kaplan-Meir method and compared by log-rank test. P values ≤0.05 were considered statistically significant. All statistical analyses were performed using Graphpad prism 9.0 software.

## Results

### Screening for Selection of the Candidate Strains With Quercetin-Degrading Property

Of 48 candidate strains tested, six strains were identified to have quercetin-degrading rate more than 35%, including the *B. dorei* XR 2020 (71.2%), *L. plantarum* (55.6%), *E. faecalis* (50.3%), *B. fragilis* (48.9%), *L. paracasei* (42.5%), and *B. longum* (35.7%). Most of other strains such as *B.subtilis* (2.6%) and *A. hadrus* (0%) could not degrade quercetin (**Table S1**, **Figure 1B**).

### Anti-Influenza Activity of the Candidate Strains

To determine whether quercetin-degrading strains could protect the host against influenza infection, we evaluated the anti-influenza efficacy of eight strains with different quercetin-degrading rate. Influenza-infected mice had significantly higher lung index, shorter colon length, and more weight loss compared with the non-infected mice. Among the eight strains, only *B. dorei* and *L. plantarum* significantly reversed the changes in lung index and colon length caused by influenza infection (**Figures 1C–E**).

### Effect of Oral Administration of *B. dorei* on Influenza Infection

#### Optimal Timing in Intervention With *B. dorei* to Fight Against Influenza

To determine the optimal timing of *B. dorei* administration, we treated mice with *B. dorei* prior to or post infection. Based on the results of lung index, length of colon, and weight loss, we found that pre-treatment with *B. dorei* was effective for its anti-influenza activity (**Supplementary Figures S1C–E**).

#### Effect of *B. dorei* on Survival and Health Status of Influenza-Infected Mice

To observe the effect of *B.dorei* administration on influenza infection-caused mortality, mice were infected with a lethal dose of influenza virus. We found that all 10 mice treated with PBS died within 10 days. In contrast, 6 out of 10 oseltamivir-treated mice, and 3 of 10 *B. dorei*-treated mice survived at day 14 p.i. (**Figure 2B**).

To confirm the effect of *B. dorei* on resistance to influenza infection, we challenged the mice with a non-lethal dose of influenza virus. The results showed that mice treated with *B. dorei* or oseltamivir had significant improvement in body weight, lung index, and colon length compared with those treated with PBS (**Figures 2C–E**).

#### Effect of *B. dorei* on Lung Viral Load in Influenza-Infected Mice

Both *B. dorei* and oseltamivir treatment significantly lowered viral load in the lungs of infected mice compared with those with PBS at day 3 p.i., however, *B. dorei* did not further reduce the viral load at day 7 p.i. while such effect was still present with oseltamivir treatment (**Figures 2F, G**).

#### Effect of *B. dorei* on Lung Pathology of Influenza-Infected Mice

**Figure 3:**
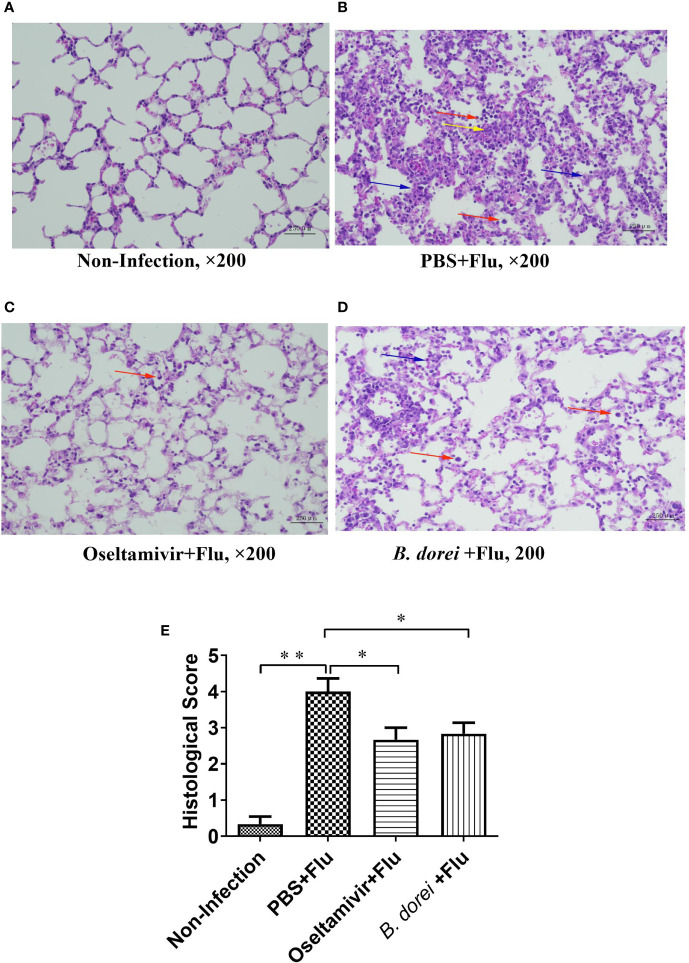
Histopathological findings of the lung of influenza-infected mice successively receiving *B. dorei* or oseltamivir at 7 day post infection. Lung histopathological examination (HE staining, 200×) are shown in panel **(A–D)**. **(A)** the control group; **(B)** the PBS+Flu group; **(C)** the *B. dorei*+Flu group; **(D)** the oseltamivir+Flu group; **(E)** Histopathological scores of four groups. Representative results are obtained from three independent experiments. *p < 0.05, **p < 0.01. Inflammatory cell infiltration (red arrows), alveolar wall thickening (blue arrows) and microvascular congestion and hemorrhage (yellow arrows).

Pathological examination of lung revealed that the infected mice treated with PBS (PBS+flu) showed significant pathological changes compared with non-infection mice (**Figures 3A, B**), while treatments with oseltamivir or *B. dorei* alleviated these changes caused by influenza infection, as evidenced by reduced alveolar wall thickening and inflammatory cell infiltration in alveoli (**Figures 3C, D**). The lung pathological scores of the infected mice treated with *B. dorei* or oseltamivir were comparable and significantly lower than that of the infected vehicle group (**Figure 3E**). Together, these results demonstrate that administration of *B. dorei* has protective effect against the influenza-induced lung damage.

### Effects of *B. dorei* Administration on Cytokine and Chemokine Profiles of Influenza-Infected Mice

**Figure 4:**
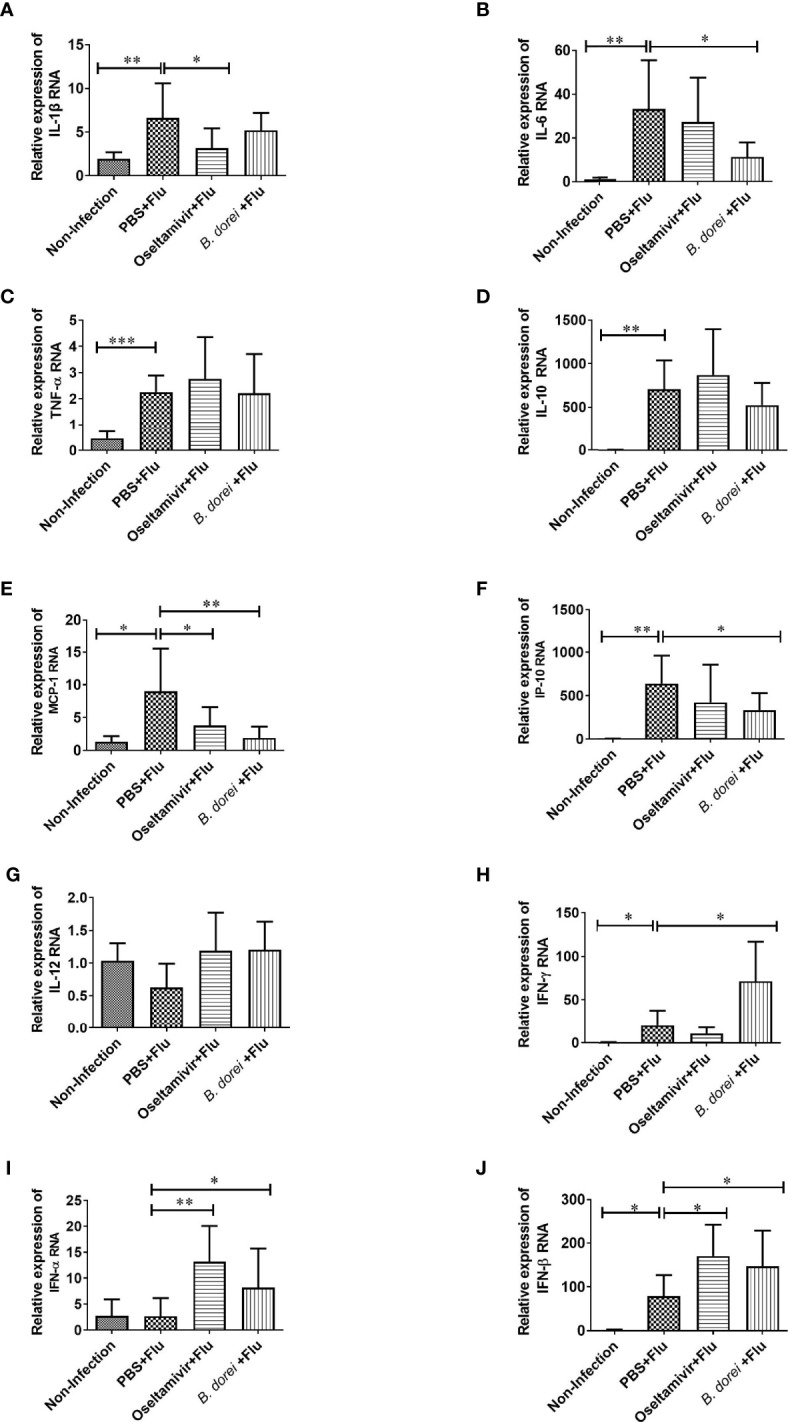
Effects of *B.dorei* on gene expressions of lung cytokine and IFNs at day 3 post infection. The gene expressions of cytokines and chemokines and IFNs of lung tissue of four groups (the Non-Infection group, the PBS+Flu group, the *B. dorei*+Flu group and the oseltamivir+Flu group) at day 3 p.i. were compared and showed as follows: IL-1β **(A)**, IL-6 **(B)**, TNF-α **(C)**, IL-10 **(D)**, MCP-1 **(E)**, IP-10 **(F)**, IL-12 **(G)**, IFN-γ **(H)**, IFN-α **(I)** and IFN-β **(J)**. The data in panels **(A–J)** are shown as mean ± standard deviation from three independent experiments. *p <0.05, **p < 0.01, ***p < 0.001.

**Figure 5:**
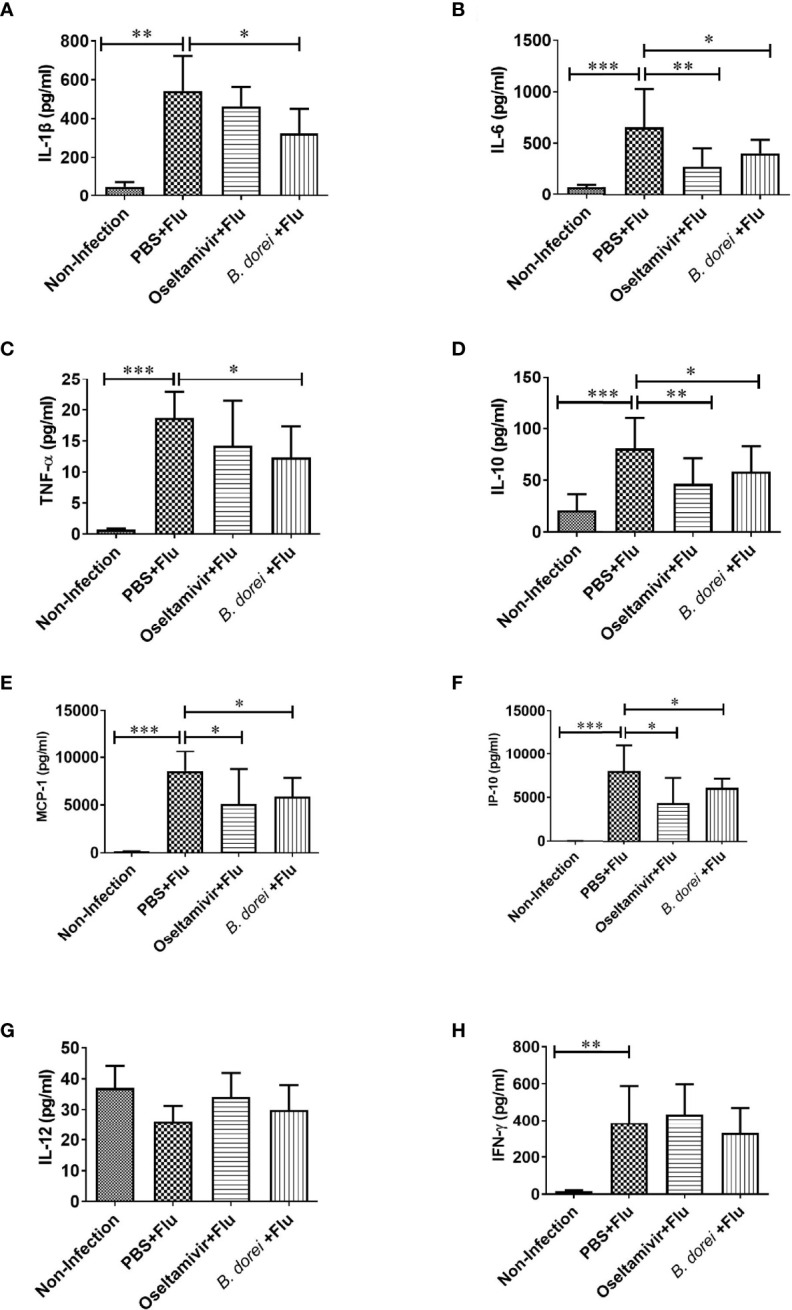
Effects of *B.dorei* on lung cytokine profiles at day 7 post infection. The levels of cytokines and chemokines in lung tissue of the Non-Infection group, the PBS+Flu group, the *B. dorei*+Flu group and the oseltamivir+Flu group were compared and showed as follows: IL-1β **(A)**, IL-6 **(B)**, TNF-α **(C)**, IL-10 **(D)**, MCP-1 **(E)**, IP-10 **(F)**, IL-12 **(G)** and IFN-γ **(H)**. The data in panels **(A–H)** are shown as mean ± standard deviation from three independent experiments. *p < 0.05, **p < 0.01, ***p < 0.001.

**Figure 6:**
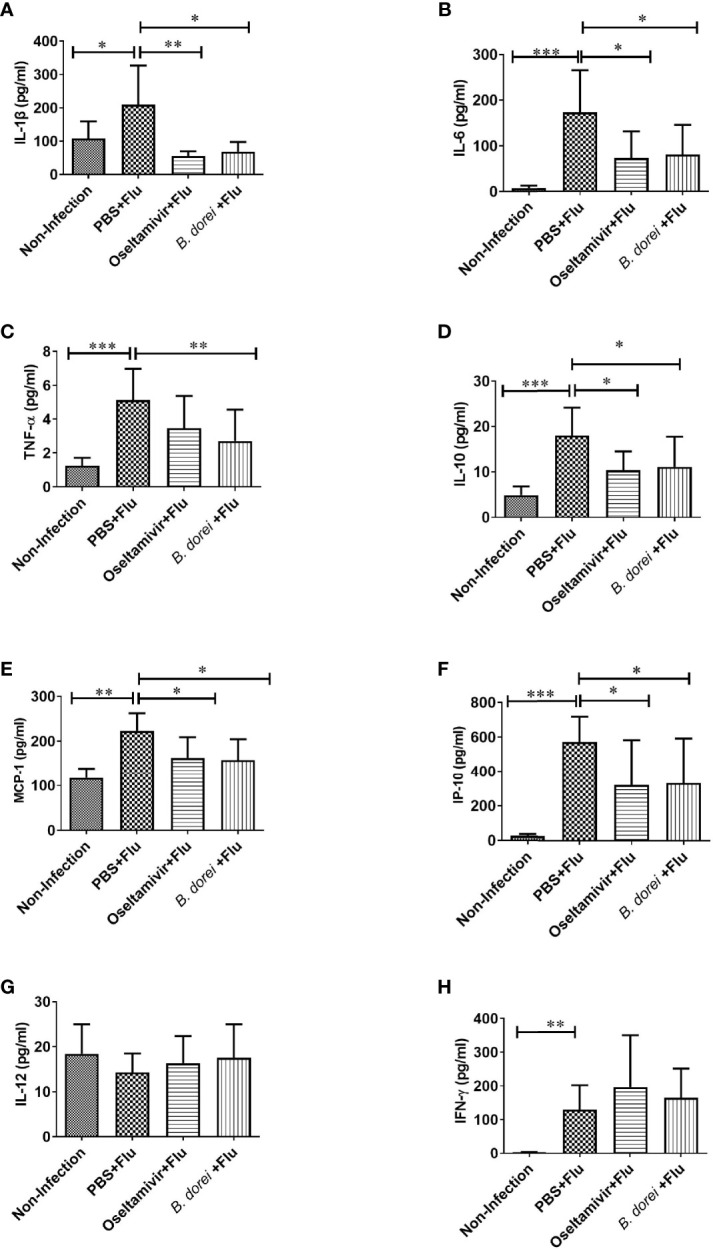
Effects of *B.dorei* on serum cytokine profiles at day 7 post infection. The serum levels of cytokines and chemokines of the Non-Infection group, the PBS+Flu group, the *B. dorei*+Flu group and the oseltamivir+Flu group were compared and showed as follows: IL-1β **(A)**, IL-6 **(B)**, TNF-α **(C)**, IL-10 **(D)**, MCP-1 **(E)**, IP-10 **(F)**, IL-12 **(G)** and IFN-γ **(H)**. The data in panels **(A–H)** are shown as mean ± standard deviation from three independent experiments. *p < 0.05, **p < 0.01, ***p < 0.001.

To understand whether *B. dorei* administration affected early inflammatory response to influenza infection, gene expression of multiple cytokines and chemokines in lung tissue was measured at day 3 p.i. using qRT-PCR (**Figure 4**). The results showed that influenza infection induced significantly higher mRNA expressions of IL-1β, IL-6, TNF-α, IL-10, MCP-1, IP-10, IFN-γ, and IFN-β in lung compared with non-infection mice (**Figures 4A–J**). *B. dorei* administration significantly reduced IL-6, MCP-1, and IP-10 expression (**Figures 4B, E, F**) and increased IFN-γ, IFN-α, and IFN-β expression compared with vehicle-treated mice (**Figures 4H–J**). We also conducted multiplex measurement on cytokines and chemokines at day 7 p.i. in lung tissues using Luminex 200, and similarly, we found significantly higher production of IL-1β, IL-6, TNF-α, IL-10, MCP-1, IP-10, and IFN-γ caused by influenza infection on day 7 p.i. (**Figures 5A–H**). Again, *B. dorei-*treatment significantly reduced the levels of IL-1β, IL-6, TNF-α, IL-10, MCP-1 and IP-10 in lung compared with PBS-treated infection mice at day 7 p.i., to a comparable degree relative to the oseltamivi treatment (**Figures 5A–F**). Concurrent with the results of lung tissue, the serum levels of IL-1β, IL-6, TNF- α, IL-10, MCP-1, IP-10, and IFN-γ, except IL-12, in infected-mice were also significantly higher than those in uninfected mice (**Figures 6A–H**). *B. dorei* administration significantly decreased serum levels of IL-1β, IL-6, TNF-α, IL-10, MCP-1, and IP-10 compared to the vehicle-treated mice (**Figures 6A–F**). These results indicate that administration of *B. dorei* protects mice against influenza infection partially due to down-regulation of both local and systemic inflammatory response and up-regulation of IFNs.

### Effect of *B. dorei* Administration on the Gut Microbiota of Influenza-Infected Mice

**Figure 7:**
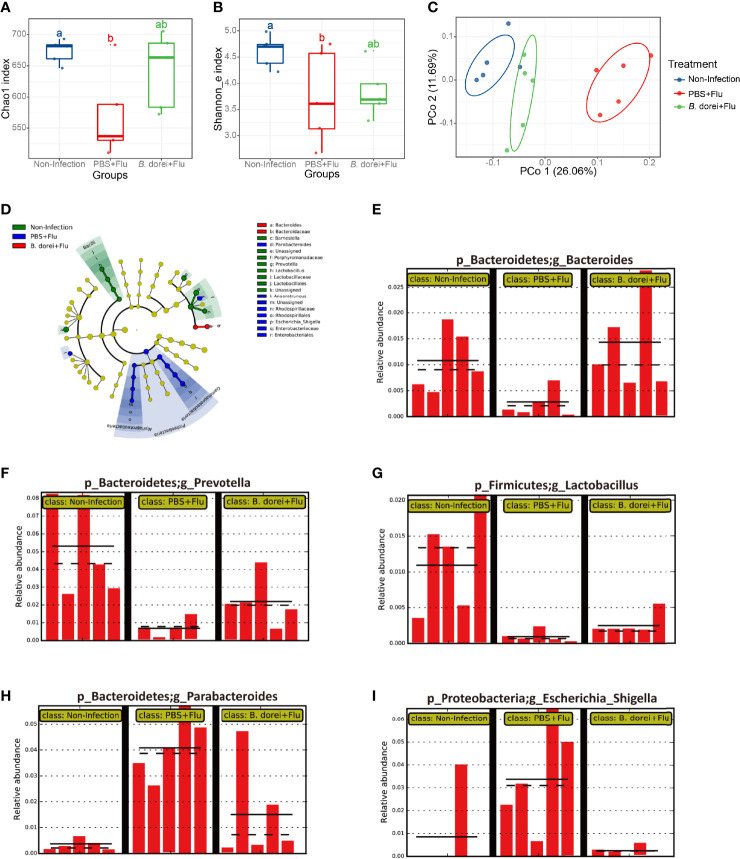
Effects of *B. dorei* on diversity and composition of gut microbiota of influenza-infected mice. **(A)** Chao1 index. **(B)** Shannon_e index. **(C)** The PCoA plot based on unweighted UniFrac metrics. **(D)** LEfSe taxonomic cladogram. **(E-I)** represent the relative abundance of the genus of Bacteroides, Prevotella, Lactobacillus, Parabacteroides, and Escherichia_Shigella by LEfSe analysis with a threshold of P = 0.05, respectively. Groups followed by the same letters are not significantly different according to Tukey’s HSD test **(A, B)**. The PCoA was compared with nonparametric MANOVA based on Adonis (P < 0.05).

The composition of the gut microbiome has been suggested to influence the immune response to influenza infection (32). To understand whether the *B. dorei* administration protects influenza infection through modulating the gut microbiota, we determined the composition of feces microbiota at day 7 p.i. using16 S rRNA sequencing. We found that both the diversity index Shannon and richness index Chao1 were significantly decreased in the infected, vehicle-treated mice compared to uninfected mice (Chao1:680± 23 VS 550± 45; Shannon: 4.6± 0.3 VS 3.5± 0.5; both at P<0.01). *B. dorei* administration partially reversed the two indexes but not reaching significant level (**Figures 7A, B**). We analyzed β-diversity based on unweighted unifrac distance and observed that three separated groups were clearly distinguished, which indicates that administration of *B. dorei* affects the structure of microbiota in influenza-infected mice (**Figure 7C**). The phylogenetic analysis demonstrated that the microbiota of three groups were located at different taxonomic clades (**Figure 7D**).

We also observed that microbiota composition at genus level was associated with *B. dorei* administration. The result of LEfSe analysis showed that the relative abundance of *Bacteroides*, *Prevotella* and *Lactobacillus* in infected, vehicle-treated mice were significantly lower than those in uninfected mice, and these infection-induced changes were reversed by *B. Dorei* treatment (**Figures 7E–G**). In contrast, the relative abundance of *Escherichia*, *Shigella* and *Parabacteroides* in the infected vehicle-treated mice were significantly higher than those in uninfected mice, while *B. dorei* treatment significantly decreased their relative abundance (**Figures 7H, I**). Together, these results demonstrate that administration of *B. Dorei* could reverse the alterations in gut microbiota composition caused by influenza infection by increasing the levels of *Bacteroides*, *Prevotella*, *Lactobacillus* while decreasing the levels of *Escherichia*, *Shigella* and *Parabacteroides*.

## Discussion

In this study we screened 48 bacteria species isolated from healthy human feces or fermented food and identified an anaerobic bacteria *B. dorei* that had robust ability to degrade quercetin (degrading rate:72.5%). Using a mouse model, we, for the first time, determined that *B. dorei* had protective effect against influenza infection.

Most of the common probiotic strains that have been reported to have protective effect against influenza infection belong to *Lactobacillus* and *Bifidobacterium* genera, such as *Lactobacillus gasseri* (5, 6), *Lactobacillus plantarum* (1, 7), *Lactobacillus rhamnosus* (8, 9), *Lactococcus lactis* (33), *Bifidobacterium longum* (12, 13) and *Lactobacillus paracasei* (11). However, one recent study reported that *Bacteroides fragilis* can induce IFN-β expression with its capsular polysaccharide A (PSA) through TLR4 receptor of the dendritic cells in colonic lamina propria and protect murine vesicular stomatitis virus (VSV)-infected mice and influenza virus-infected bone marrow-derived dendritic cells (34). Moreover, they presumed that the activity of IFN-β induction is a shared function of *Bacteroides* sp. In this study, as a member of *Bacteroides* genus, *B. dorei* XR2020 strain was found to improve survival, reduce viral load, and rapidly up-regulate IFNs expression in lungs at day 3 p.i., which is consistent with findings in previous studies (23, 34). Several probiotic strains have anti-inflammatory activity and exert protection against influenza infection in mice with accompanied changes in Th1 and Th2 cytokines. The findings in this study, i.e., enhanced Th1 cytokine responses and suppressed Th2-mediated immune response by *B. dorei* treatment, were largely consistent with most but not all the previous studies as there exist some conflicting data for particular cytokines among these studies. Little is known regarding the effect of *B. dorei* in modulating inflammatory response of immune cells. One study reported that *B. dorei* markedly increased abundance at time of onset of type I diabetes. Exposure of isolated human islets to *B. dorei* revealed the upregulated *CHAC1*. Upregulation of *CHAC1* is proinflammatory event and it mediates tissue inflammation through controlling the release of cytokines (35). In this context, it is noteworthy that the higher and earlier response of type I IFNs and IFN-γ induced by *B. dorei* may play a key role in restricting virus replication in lung, which may result in a shorter illness course and alleviated lung pathological damage in influenza infection.

It has been reported that people infected with influenza have higher levels of cytokines stimulated by type I IFNs (such as IL-6 and IFN-γ), and the chemokines IP-10 (also known as CXCL-10) and MCP-1 (CCL2) (36–38). While type I IFNs are critical in body’s antiviral responses, they also contribute to pathological consequence, particularly in the acute and severe influenza infection (39). Type I IFN-induced inflammatory response in patients with severe influenza are believed to play a pivotal role in exacerbating inflammation (40). Higher level of IFNs and their downstream cytokines in late infection may accelerate excessive inflammatory responses. In this study, we observed that *B.dorei* administration decreased production of several cytokines (IL-1β, IL-6, IL-10,TNF-α, MCP-1, and IP-10) in lung and serum induced by influenza infection, which may alleviate excessive inflammatory response and ameliorate immunopathologic damage. These results suggest that the protective effect of *B. dorei* against influenza infection may be in part mediated through down-regulating several inflammatory cytokines and chemokines. We also observed that *B. dorei*-induced higher expression of IFN-γ was only observed at day 3 p.i. but not at day 7 p.i. compared with control infected mice, which suggests that *B. dorei’s* anti-influenza effect may be related to its timely action in enhancing antiviral response in the early stage of infection while preventing excessive and long-lasting inflammatory damage.

The increasing evidence indicates that alterations of gut microbiota may affect pulmonary immunity relevant to viral infections (41). Emerging experimental data highlight a crucial crosstalk between the gut microbiota and the lungs, termed as the gut-lung axis (42, 43). The immune responses in the gut-lung axis depend on balanced gut microbiota composition (44). Two recent studies showed that opportunistic pathogens like *Actinomyces, Erysipelaclostridium, Streptococcus, Veillonella, Rothia*, and *Enterobacter* were associated with COVID-19 diagnosis and/or severity of infection; conversely, beneficial butyrogenic bacteria like *Faecali bacterium*, *Anaerostipes*, and *Bifidobacterium* were inversely correlated with COVID-19 diagnosis and/or severity (28, 45). We found that influenza infection caused reduction of beneficial bacteria (*Bacteroides*, *Prevotella* and *Lactobacillus)* and increase of harmful bacteria (*Escherichia*, *Shigella* and *Parabacteroides*), and this influenza infection-induced alteration in microbiota composition was restored by administration of *B. dorei*. However, Belkacem et al. reported that consumption of *Lactobacillus paracasei* improved health status of influenza-infected mice but did not reverse the gut microbiota (11). This discrepancy implies that different probiotic strains may protect host against influenza through different mechanisms.

The metabolites generated by intestinal microbiota, such as desaminotyrosine (DAT) and short chain fatty acids, can interact with host to exert immunoregulatory effects (42, 43). Steed et al. reported that *C. orbiscindens* protected mice against influenza by metabolizing quercetin substrate to DAT. Administration of DAT prior to influenza infection effectively prevented the mice from infection by enhancing the type I IFN signaling but administration of DAT 2 days post infection led to a worse outcome due to the excessive inflammatory pathological damage caused by delayed type I IFN response (23). In our study administration of *B. dorei* either prior to or post infection provided similar protection to mice infected with influenza, which implicates that *B. dorei* is safe to use and effective for both preventive and therapeutic purposes.

In conclusion, in this study we demonstrated that administration of *B. dorei*, an intestinal anaerobic bacteria with a robust ability of degrading quercetin, can provide an effective protection against influenza. The mechanisms underlying this effect may involve *B. dorei*’s action in enhancing earlier interferon expression, modulating balance of pro- and anti-inflammatory cytokines, and restoring the composition of gut microbiota. Thus, *B. dorei* may be considered as a novel candidate of probiotics for potential clinical application in preventing and treating influenza infection. Considering the fact that *B. dorei*’s effect on earlier interferon expression was not specific to influenza virus, and also the recent report showing that *B. dorei* were negatively correlated with SARS-CoV-2 load in fecal samples of hospitalized COVID-19 patients, we speculate that *B. dorei* may be effective in fighting against respiratory infection caused by a broader spectrum of viruses.

## Data Availability Statement

The datasets presented in this study can be found in online repositories. The names of the repository/repositories and accession number(s) can be found below: https://www.ncbi.nlm.nih.gov/, PRJNA769975.

## Ethics Statement

The animal study was reviewed and approved by the Laboratory Animal Welfare and Ethics Committee of the National Institute for Communicable Disease Control and Prevention, Chinese Center for Disease Prevention and Control.

## Author Contributions

LS and ZR conceptualized the study, LS, YH, GL, XL, YX, CL, YZ, and JL conducted the experiments, LS and YH performed data analysis, LS and ZR prepared the paper. All authors contributed to the article and approved the submitted version.

## Funding

This work was supported by a grant from Research Units of Discovery of Unknown Bacteria and Function (2018 RU010), and Ministry of Science and Technology of China (Grant No. 2018ZX10301403-003-003).

## Conflict of Interest

The authors declare that the research was conducted in the absence of any commercial or financial relationships that could be construed as a potential conflict of interest.

## Publisher’s Note

All claims expressed in this article are solely those of the authors and do not necessarily represent those of their affiliated organizations, or those of the publisher, the editors and the reviewers. Any product that may be evaluated in this article, or claim that may be made by its manufacturer, is not guaranteed or endorsed by the publisher.

## Supplementary Material

Supplementary Figure S1 Effect of *B.dorei* administration before or after infection on against influenza infection. **(A, B)** shows the schedule of animal experiment 2. Twenty-four 6-week old female C57BL/6J mice were randomly allocated to 4 groups with six mice per group: Non-Infection group, PBS+Flu group, pre-treated *B.dorei* +Flu group and post-treated *B.dorei* +Flu group. **(C)** The comparison of the lung index among the four groups. **(D)** The comparison of colon length among the four groups. **(E)** The comparison of body weight loss among the four groups. Flu, influenza virus. PBS, phosphate-buffered saline. The data in panels **(C–E)** are shown as mean ± standard deviation from three independent experiments. *p < 0.05, **p < 0.01.

Supplementary Figure S2 Effect of alive or pasteurized *B. dorei* administrations on mice against influenza infection.Thirty 6-week old female C57BL/6J mice were randomly allocated to 5 groups with six mice per group: Non-Infection group, PBS+Flu group, Oseltamivir+Flu group, Alive *B.dorei* +Flu group and pasteurized *B. dorei* +Flu group. **(A)** shows the survivals 14 days post infection (p.i.) of five groups in animal experiment; **(B)** shows the dynamic trend in the body weight of the mice in each group after infection; **(C)** shows the lung index of the mice in each group at day 7 p.i.; **(D)** shows the colon length of the mice in each group at day 7p.i.; **(E)** shows the viral load in lung tissue at 3 p.i.; **(F)** shows the viral load in lung tissue at 7 p.i.. The data in panels **(A, B)** are obtained from one of three independent experiments. The data in panels **(C–F)** are shown as mean ± standard deviation from three independent experiments. *p < 0.05, **p < 0.01, ***p < 0.001.
