# Supplementary figures and images for: A Novel Immunobiotics Bacteroides dorei Ameliorates Influenza Virus Infection in Mice
Source: Front Immunol. 2022 Jan 26;12:828887. doi: 10.3389/fimmu.2021.828887 (PMC8826429; doi:10.3389/fimmu.2021.828887)

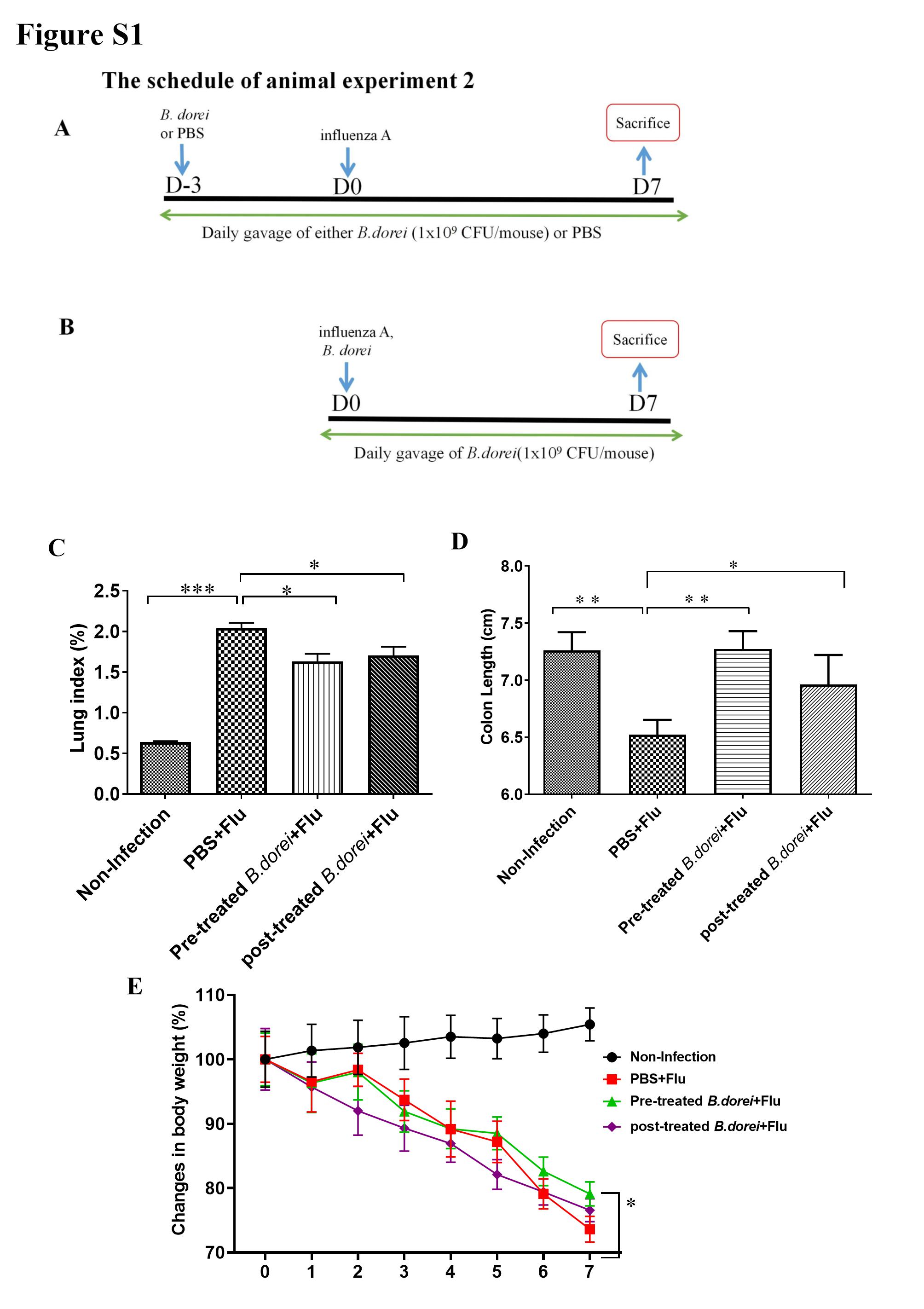

Supplement: Supplementary Figure S1 — Effect of B.dorei administration before or after infection on against influenza infection. (A, B) shows the schedule of animal experiment 2. Twenty-four 6-week old female C57BL/6J mice were randomly allocated to 4 groups with six mice per group: Non-Infection group, PBS+Flu group, pre-treated B.dorei +Flu group and post-treated B.dorei +Flu group. (C) The comparison of the lung index among the four groups. (D) The comparison of colon length among the four groups. (E) The comparison of body weight loss among the four groups. Flu, influenza virus. PBS, phosphate-buffered saline. The data in panels (C–E) are shown as mean ± standard deviation from three independent experiments. *p < 0.05, **p < 0.01. [file Image_1.jpeg]

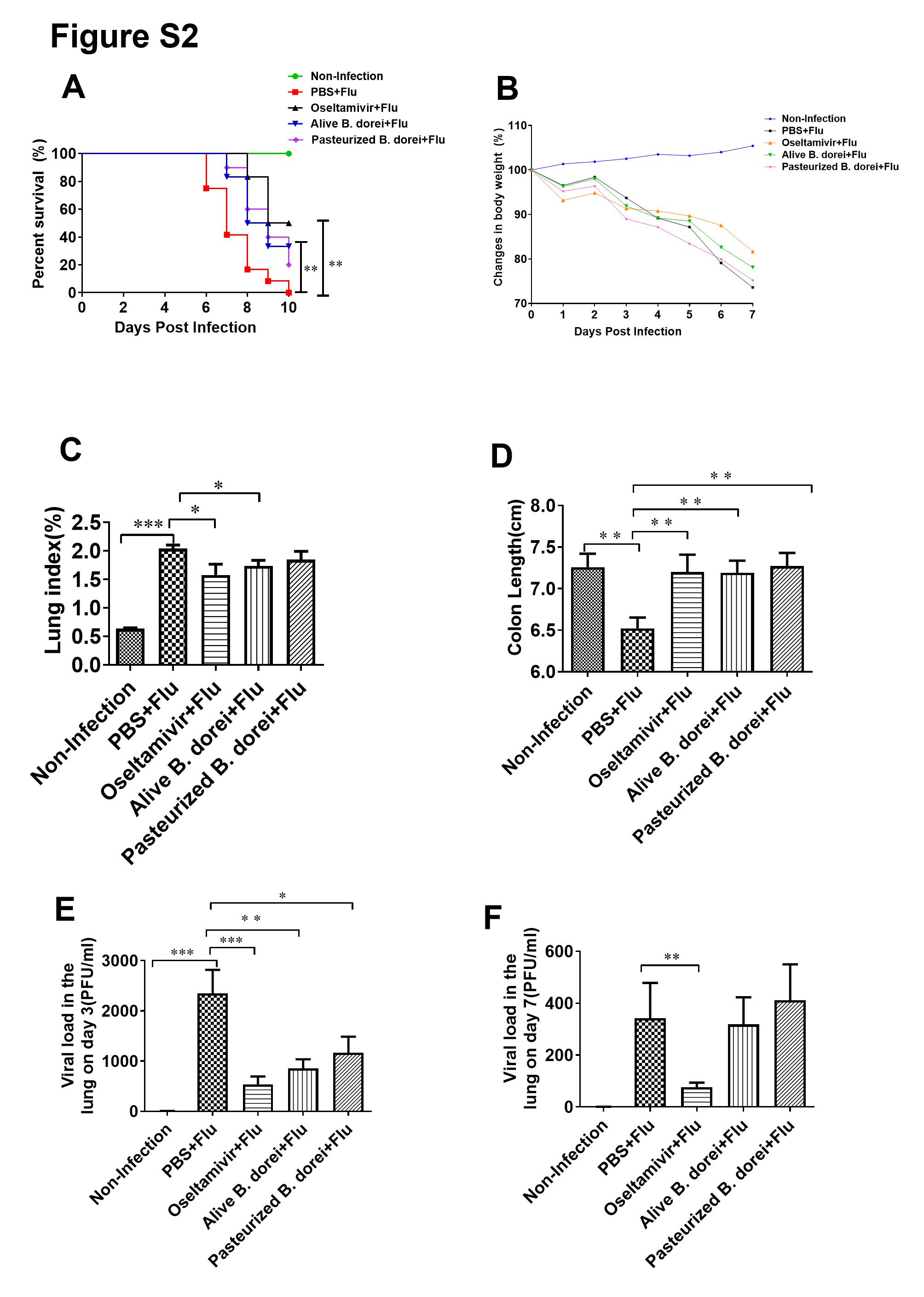

Supplement: Supplementary Figure S2 — Effect of alive or pasteurized B. dorei administrations on mice against influenza infection.Thirty 6-week old female C57BL/6J mice were randomly allocated to 5 groups with six mice per group: Non-Infection group, PBS+Flu group, Oseltamivir+Flu group, Alive B.dorei +Flu group and pasteurized B. dorei +Flu group. (A) shows the survivals 14 days post infection (p.i.) of five groups in animal experiment; (B) shows the dynamic trend in the body weight of the mice in each group after infection; (C) shows the lung index of the mice in each group at day 7 p.i.; (D) shows the colon length of the mice in each group at day 7p.i.; (E) shows the viral load in lung tissue at 3 p.i.; (F) shows the viral load in lung tissue at 7 p.i.. The data in panels (A, B) are obtained from one of three independent experiments. The data in panels (C–F) are shown as mean ± standard deviation from three independent experiments. *p < 0.05, **p < 0.01, ***p < 0.001. [file Image_2.jpeg]
